# The relationship between esophageal acidity and symptom frequency in symptomatic nonerosive gastroesophageal reflux disease

**DOI:** 10.14814/phy2.15442

**Published:** 2022-08-27

**Authors:** Jerry D. Gardner

**Affiliations:** ^1^ Science for Organizations Mill Valley California USA

**Keywords:** interval esophageal acid exposure time, interval esophageal acidity, nonerosive gastroesophageal reflux disease

## Abstract

The present paper examines the extent to which novel measures of esophageal acid exposure can elucidate possible relationships between symptom perception and esophageal acidity in subjects with nonerosive gastroesophageal reflux disease. Recordings of esophageal pH and symptom occurrence from 20 subjects with nonerosive gastroesophageal reflux disease were analyzed. Interval esophageal acid exposure was calculated in two different ways for the interval that preceded each symptom in each subject. Interval esophageal acidity was calculated as the time‐weighted acid concentration for the interval. Interval esophageal acid exposure time was calculated as the percentage of the total recording time that esophageal pH was less than pH 4 for the interval. There was a negative relationship between the probability of a symptom and interval esophageal acid exposure indicating the paradoxical finding that the lower the value of esophageal acid exposure, the higher the probability of a symptom. The time courses of symptoms and cumulative esophageal acidity resolved this paradox by indicating that esophageal acid exposure oscillates between longer periods of low esophageal acid exposure with a high number of symptoms reflecting high esophageal acid sensitivity, and shorter periods of high esophageal acid exposure with fewer symptoms reflecting low esophageal acid sensitivity. Thus, the present analyses show how novel measures of acidity can identify and also resolve a previously unrecognized paradoxical relationship between esophageal acid exposure and symptom frequency in subjects with nonerosive gastroesophageal reflux disease.

## Graphical abstract

The present paper examines the extent to which novel measures of esophageal acid exposure can elucidate possible relationships between symptom perception and esophageal acidity in subjects with nonerosive gastroesophageal reflux disease. There was a negative relationship between the probability of a symptom and interval esophageal acid exposure indicating the paradoxical finding that the lower the value of esophageal acid exposure, the higher the probability of a symptom. The time courses of symptoms and cumulative esophageal acidity resolved this paradox by indicating that esophageal acid exposure oscillates between longer periods of low esophageal acid exposure with a high number of symptoms reflecting high esophageal acid sensitivity, and shorter periods of high esophageal acid exposure with fewer symptoms reflecting low esophageal acid sensitivity.

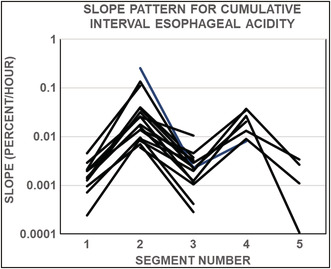

## 1 INTRODUCTION

Recently, the Lyon Consensus Conference (Gyawali et al., 2018) proposed criteria for the clinical diagnosis of gastroesophageal reflux disease (GERD) and commented that the association of esophageal acid exposure and symptoms of GERD is weak. Nearly, all previous analyses of relationships between GERD symptoms and esophageal acidity have used the symptom index (SI; Weiner et al., 1988), or the symptom association probability (SAP; Weusten et al., 1994), which assesses the relationship between symptoms and esophageal reflux episodes identified initially by a decrease in esophageal pH to below pH 4 or more recently, by a change in esophageal impedance (Kamal et al., 2020). The SI and SAP are standard measures of impedance–pH studies, and have been used clinically to identify different phenotypes of symptomatic GERD subjects (Gyawali et al., 2018).

For the present analyses, however, I was interested in possible relationships between esophageal acid exposure and symptom frequency in subjects with symptomatic nonerosive gastroesophageal reflux disease (NERD). I calculated interval esophageal acid exposure from the time of one symptom until the time of the next symptom using two different methods—interval esophageal acidity (Gardner et al., 2001) and interval esophageal acid exposure time (Gyawali et al., 2018). Thus, each symptom is associated with two particular values for esophageal acid exposure. The entire interval between symptoms instead of just a portion of it was chosen because there was no apparent reason to do otherwise. Also, previous analyses have found that there is a statistical association between sequential values of esophageal pH for up to 2 h in normal as well as GERD subjects (Gardner et al., 2005); therefore, it seemed appropriate to capture information from the entire inter‐symptom interval.

## 2 METHODS

Subjects were identified for the present analyses by interrogating the electronic database (January 2016–August 2019) at the Royal London Hospital GI Physiology Unit. All subjects in the Royal London Hospital database had undergone impedance–pH monitoring because the referring physician reported an unsatisfactory response to gastric antisecretory medication such as proton pump inhibitors (PPIs). No details regarding the unsatisfactory response were provided by the referring physician. Subjects were included in the database if they were over 16 years old and underwent upper gastrointestinal endoscopy, high‐resolution manometry (HRM), and 24‐h impedance–pH monitoring while not taking a PPI. Patients were excluded if they had (i) endoscopic esophagitis, Barrett's esophagus, or eosinophilic esophagitis, (ii) HRM diagnosis of a major esophageal motility disorder, or (iii) belching as the main symptom.

All subjects had undergone the following procedures. HRM (ManoScan, Medtronic) and impedance–pH monitoring (Sandhill Scientific) were performed after overnight fasting. Ambulatory impedance–pH monitoring was performed following HRM with gastric antisecretory agents having been discontinued for at least 7 days. The impedance–pH catheter was inserted with an esophageal pH sensor positioned 5 cm above the upper border of the lower esophageal sphincter, and a gastric pH sensor positioned 15 cm below the esophageal pH sensor. Six impedance channels were positioned 3, 5, 7, 9, 15, and 17 cm above the lower esophageal sphincter, respectively. The pH electrodes were calibrated following the instructions from the manufacturer and using their standard calibration solutions at pH 4 and pH 7. Because antimony electrodes, which are temperature sensitive, were used to record pH, the software provided by Sandhill to process pH recordings automatically adjusts all pH values for the difference between the calibration temperature 25°C and the recording temperature 37°C. Software provided by the manufacturer was also used to export pH data for every 4th second of the recording to a Microsoft Excel file. Values of pH below 0.5 were replaced with 0.5, and pH values above 7.5 were replaced with 7.5.

Subjects were instructed to follow their usual daily routines and meals, but to avoid carbonated or acidic beverages. Subjects pressed an event marker to signal the meal periods, recumbent period, and symptoms of heartburn, regurgitation, or chest pain.

Recordings from 20 subjects with NERD defined as acid exposure time >6% regardless of values for SI and SAP (Gyawali et al., 2018) were selected for analysis. Table A1 in Appendix gives traditional values for impedance–pH testing in the NERD subjects selected for analysis.

For this retrospective analysis of clinically indicated tests with no identifiable patient data, formal ethics approval was not deemed necessary.

**FIGURE 1:**
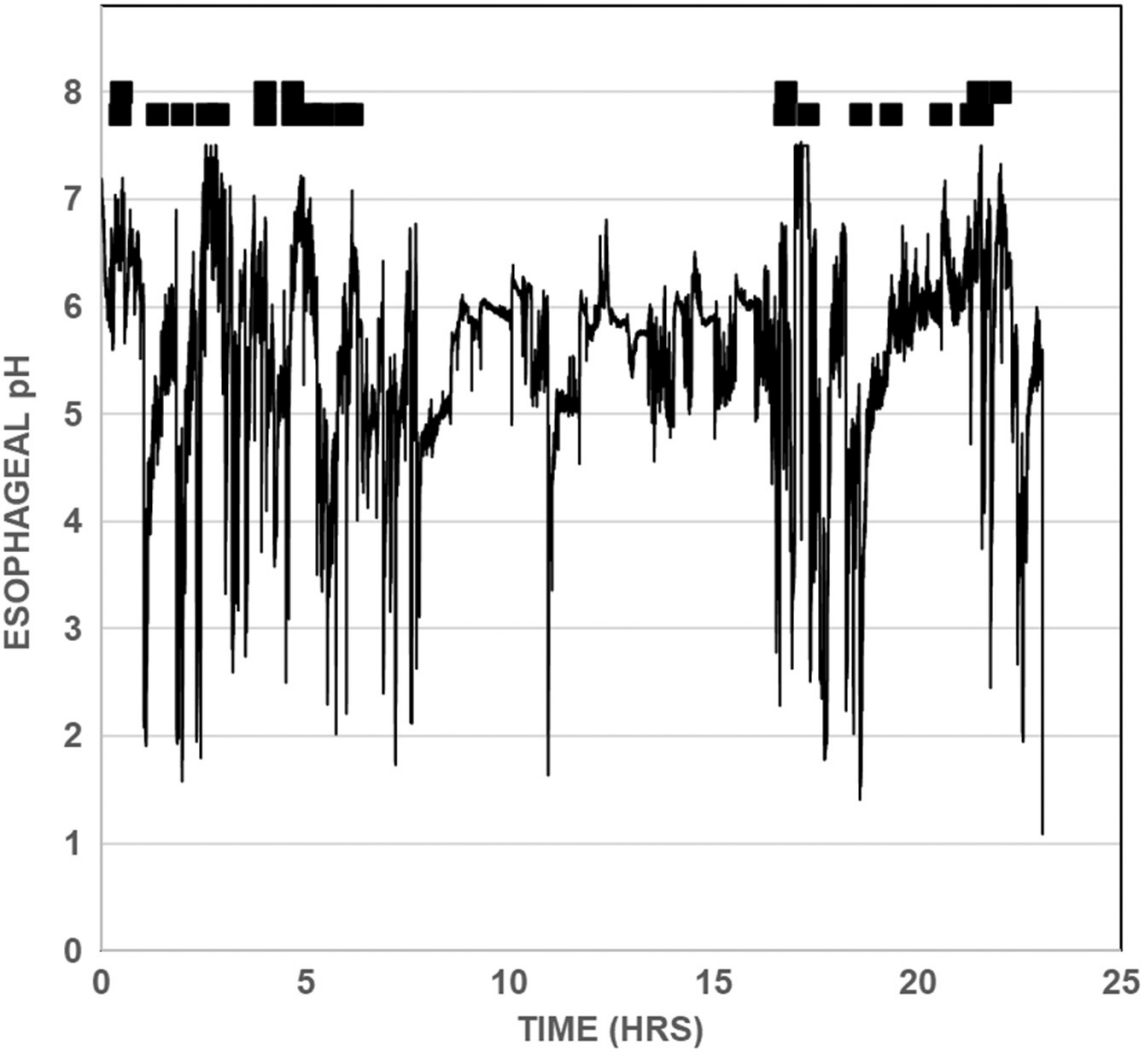
Esophageal pH recording from a nonerosive gastroesophageal reflux disease subject. Solid symbols at the top of the figure indicate times at which the subject reported a symptom.

Figure 1 illustrates a representative esophageal pH recording and the time at which each of 25 symptoms occurred. Symptoms appeared to occur in two different groups. One group of symptoms occurred during the first 6 h of the recording and the second group occurred during the last 5 h of the recording.

**FIGURE 2:**
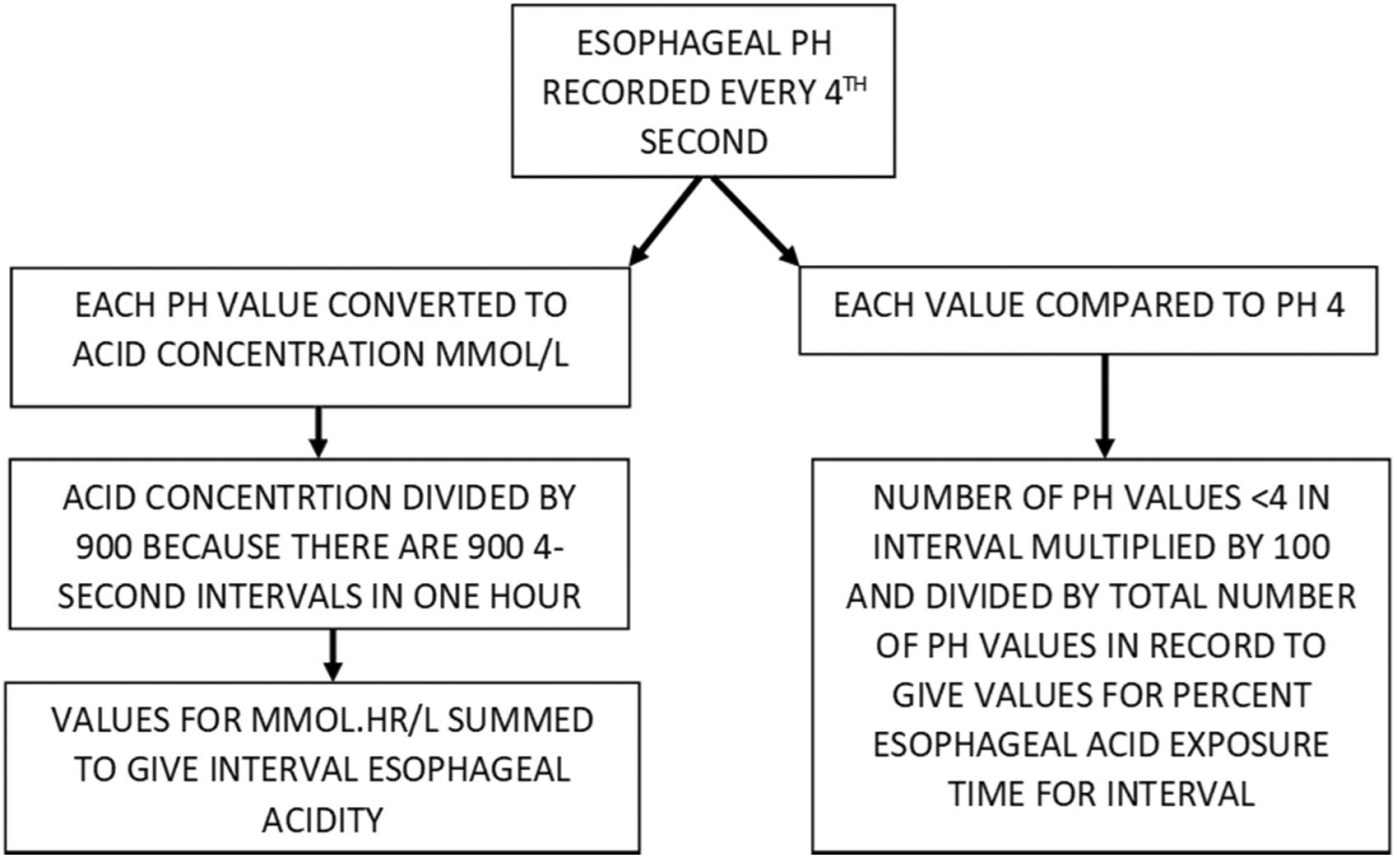
Schematic diagram illustrating the procedures for calculating interval esophageal acidity and interval esophageal acid exposure time. In each subject, interval esophageal acidity and interval esophageal acid exposure time were calculated from the beginning of the pH recording until the time of the first symptom, from the time of the first symptom until the time of the second symptom, and so on until the time of the last symptom,

Esophageal acid exposure time measured as the percentage of total recording time that esophageal pH is less than pH 4 is what most investigators use to measure esophageal acid exposure (Gyawali et al., 2018; Table A1 in Appendix). Figure 2 illustrates that for each esophageal pH recording, interval esophageal acid exposure time was measured as the percentage of total recording time that esophageal pH was less than pH 4 and interval esophageal acidity (Gardner et al., 2001) was measured as the time‐weighted acid concentration in mmol/L from the beginning of the recording until the time of the first symptom, from the time of the first symptom until the time of the second symptom and so on until the time of the last symptom. These procedures result in each symptom being associated with a specific value of interval esophageal acidity and interval esophageal acid exposure time.

Cumulative interval esophageal acidity and cumulative interval esophageal acid exposure time for the time course for each subject were calculated as the sum of sequential values of interval acidity and acid exposure time for each symptom until the time of the last symptom.

Curve fitting and statistical analyses were performed using GraphPad Prism software.

## 3 RESULTS

NERD subjects (9 males; 11 females) ranged in age from 22 to 74 years. The median number of symptoms was 24 with an interquartile range of 10 to 31. Symptom distribution was heartburn 426; regurgitation 35; and chest pain 15.

Table 1 gives the time of symptoms illustrated in Figure 1 as well as the duration of the time between sequential symptoms. Values for interval esophageal acid exposure time and interval esophageal acidity associated with each symptom are also given. There was a broad range for each set of values. The time between symptoms ranged from 0.01 to 10.59 h. Interval acid exposure time ranged from 0.00% to 1.64% and interval esophageal acidity ranged from 0.00001 to 1.24 mmol·h/L.

**TABLE 1:** Values for time of symptoms and accompanying values of interval duration, interval esophageal acid exposure time, and interval esophageal acidity for the esophageal pH recording in Figure 1

Symptom number	Symptom time (h)	Interval duration (h)	Interval AET (%)	Interval ESO acidity (mmol·h/L)
1	0.44	0.44	0.00	0.00026
2	0.47	0.03	0.00	0.00001
3	1.37	0.90	0.48	0.55015
4	1.98	0.61	0.45	0.58619
5	2.58	0.61	0.65	0.39918
6	2.85	0.26	0.00	0.00004
7	3.99	1.15	0.72	0.08430
8	4.00	0.01	0.00	0.00001
9	4.67	0.67	0.30	0.02112
10	4.68	0.01	0.00	0.00001
11	4.70	0.02	0.00	0.00002
12	5.12	0.41	0.00	0.00012
13	5.43	0.31	0.17	0.01142
14	5.97	0.54	0.51	0.09085
15	6.13	0.17	0.04	0.01600
16	16.72	10.59	1.03	0.48270
17	16.76	0.04	0.00	0.00027
18	17.32	0.55	0.37	0.03409
19	18.59	1.27	1.65	1.23879
20	19.34	0.76	0.64	1.00073
21	20.56	1.22	0.00	0.00148
22	21.30	0.74	0.00	0.00047
23	21.49	0.19	0.00	0.00022
24	21.57	0.08	0.00	0.00001
25	22.02	0.45	0.17	0.01674

Abbreviation: AET, esophageal acid exposure time.

Twelve of the values for interval acid exposure time were zero indicating no values <pH 4 during the interval. Seven of these values occurred during the initial 6 h of the recording and five of these values occurred during the last 5 h of the recording. The duration of the interval during which no pH values were <pH 4 ranged from 0.01 to 1.22 h, and the accompanying value of interval esophageal acidity ranged from 0.00001 to 0.00148 mmol·hr/L.

**FIGURE 3:**
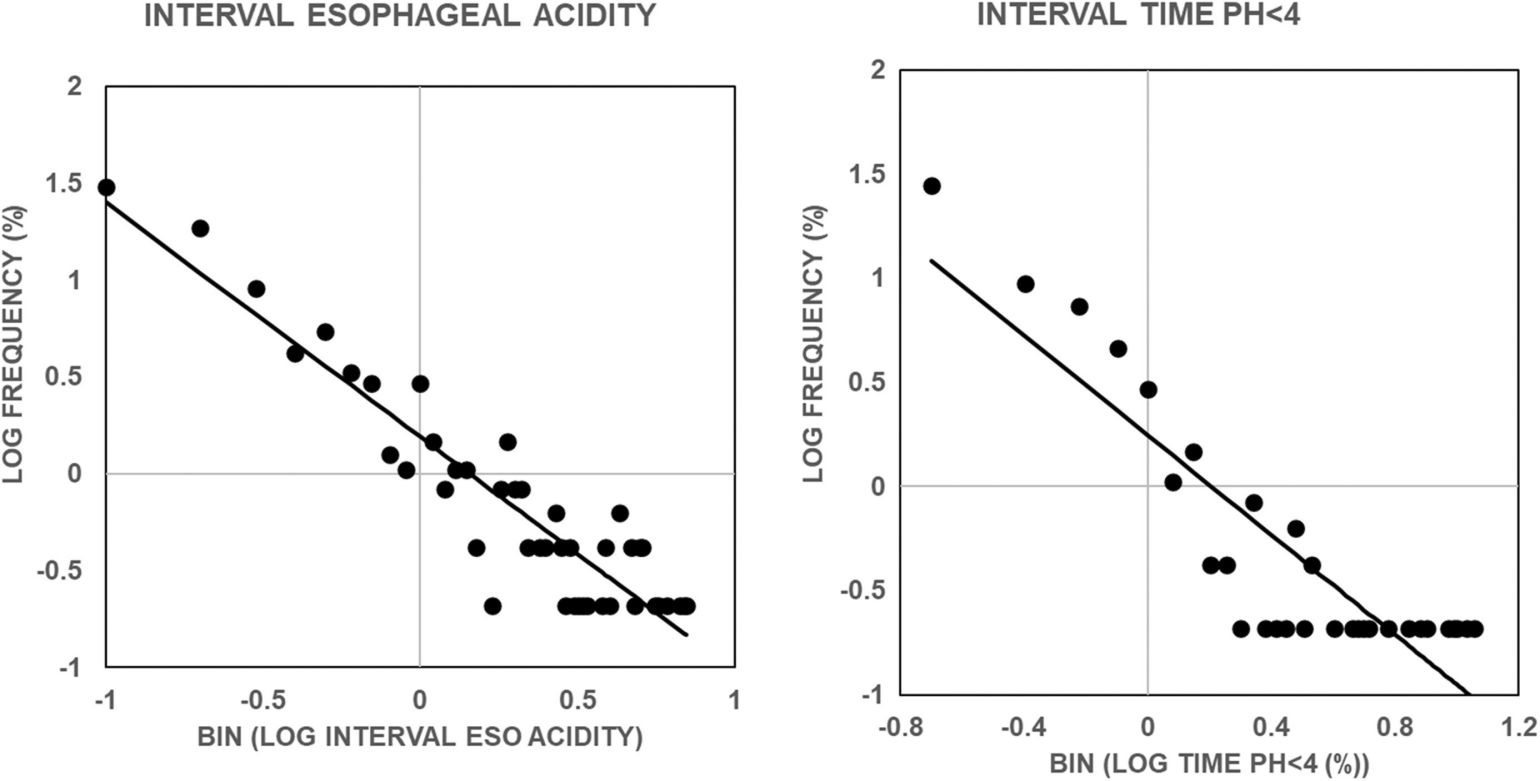
Distribution of values for interval esophageal acidity (left panel) and interval esophageal exposure time (right panel). Each distribution used a bin width of 0.5 and values given on the *x*‐axis are for the lower boundary of the bin. Values are for 476 symptoms from 20 nonerosive gastroesophageal reflux disease subjects. Every subject reported at least three symptoms during the pH recording. The solid line in each panel is the linear, least‐squares fit of the data and was significantly different from zero *p* < 0.0001 by an *F* test.

Figure 3 illustrates that the lower the bin interval, the higher the percentage of values in the bin for both interval esophageal acidity and interval esophageal acid exposure time. Since each value of esophageal acidity and esophageal acid exposure time is associated with a symptom, the percentage of values in each bin gives the percentage of total symptoms associated with that bin. Thus, Figure 3 illustrates the paradoxical finding that the lower the value of esophageal acid exposure, the higher the probability of a symptom.

**FIGURE 4:**
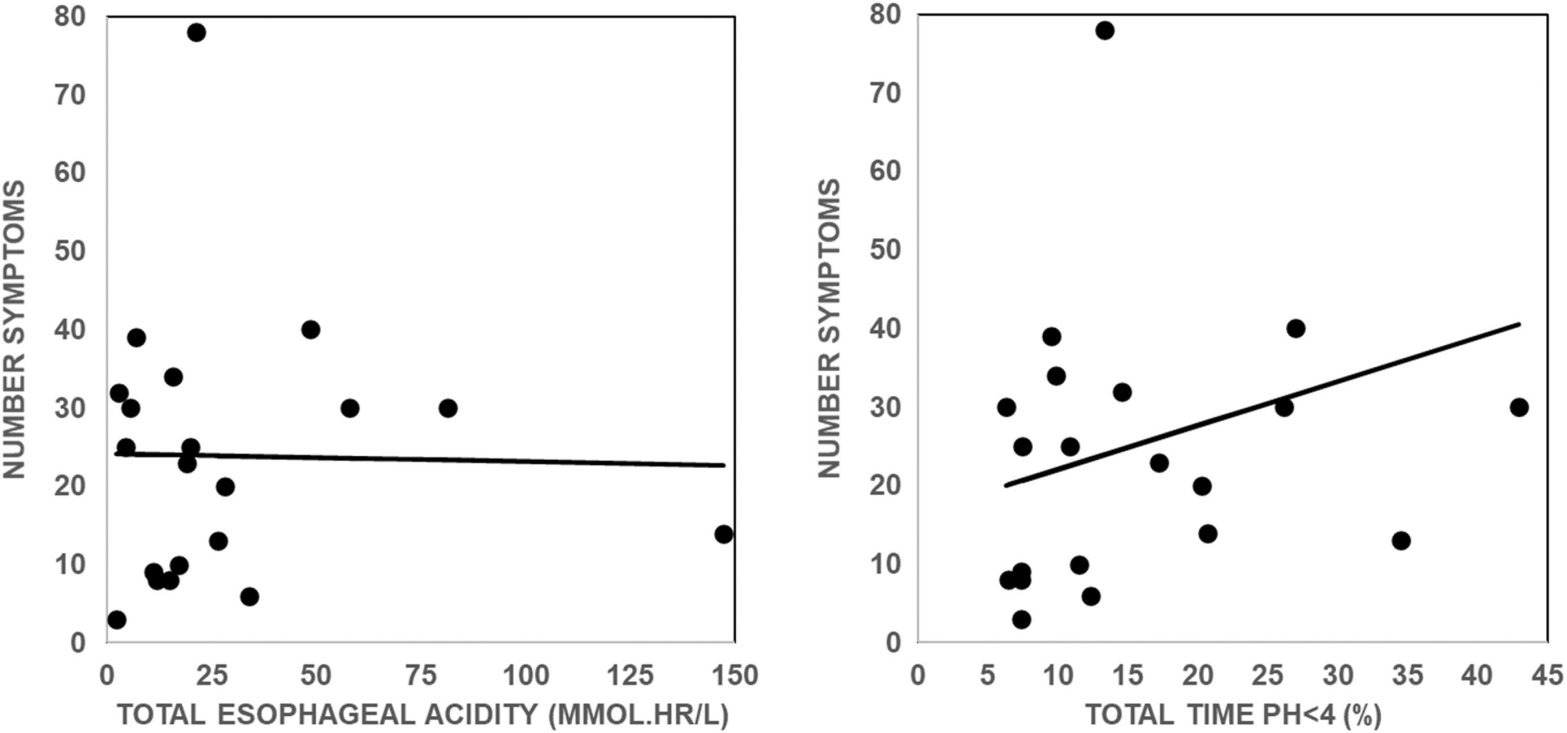
Value for total number of symptoms versus corresponding value of total esophageal acidity (left panel) and total esophageal acid exposure time (right panel). Values are from 20 nonerosive gastroesophageal reflux disease subjects. The solid line in each panel is the linear, least‐squares fit of the data and was not significantly different from zero by an *F* test (*p* = 0.9336 left panel; *p* = 0.3225 right panel).

The wide range of values for interval acidity and interval acid exposure time illustrated in Figure 3 suggests that there will not be a significant relationship between the total number of symptoms in a given subject and corresponding values for total esophageal acidity or total esophageal acid exposure time. Figure 4 examines this possibility.

Figure 4 shows that there is no significant relationship between total number of symptoms in a given subject and corresponding values tor total esophageal acidity and total esophageal acid exposure time as would be expected from the wide range of values for interval esophageal acid exposure is illustrated in Figure 3.

**FIGURE 5:**
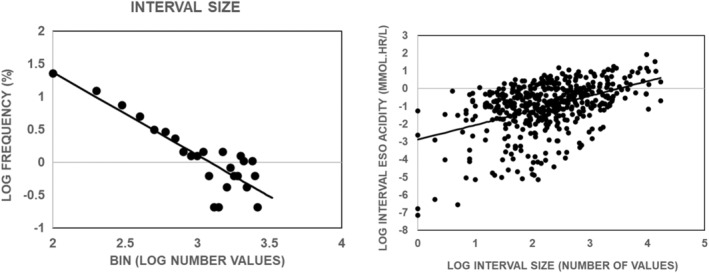
Distribution of values for interval size expressed as the number of values in the interval (left panel) and interval esophageal acidity versus interval size (right panel). The solid line in each panel is the linear, least‐squares fit of the data and was significantly different from zero *p* < 0.0001 by an *F* test.

Figure 5‐left illustrates that the lower the bin interval, the higher the percentage of values in the bin for interval size. Since each value of interval size is associated with a symptom, the percentage of values in each bin gives the percentage of total symptoms associated with that bin. Thus, Figure 5‐left illustrates that the lower the value of interval time, the higher the probability of a symptom, and the higher the value of interval time, the lower the probability of a symptom. Taken together, Figures 3 and 5‐left illustrate that the shorter the interval between symptoms and the lower esophageal acid exposure during the interval, the higher the probability of a symptom, and the longer the interval between symptoms and the higher esophageal acid exposure during the interval, the lower the probability of a symptom.

Figure 5‐right also illustrates a direct relationship between interval esophageal acidity and interval time and raises the possibility that the values for interval esophageal acidity are actually caused by the duration of the interval between symptoms. For example, during sleep, a subject might have ongoing esophageal acid exposure but no symptom so that the next symptom after the sleep period would have a high value for interval esophageal acidity. On the other hand, ingestion of a meal or changing position might cause symptoms to occur close together in time and be associated with low values of interval esophageal acidity. To examine the possibility that values for interval esophageal acidity are actually determined by the duration of the interval between symptoms, I calculated interval esophageal acidity for a fixed, 60‐s interval (15 pH values) before each of the 476 symptoms.

**FIGURE 6:**
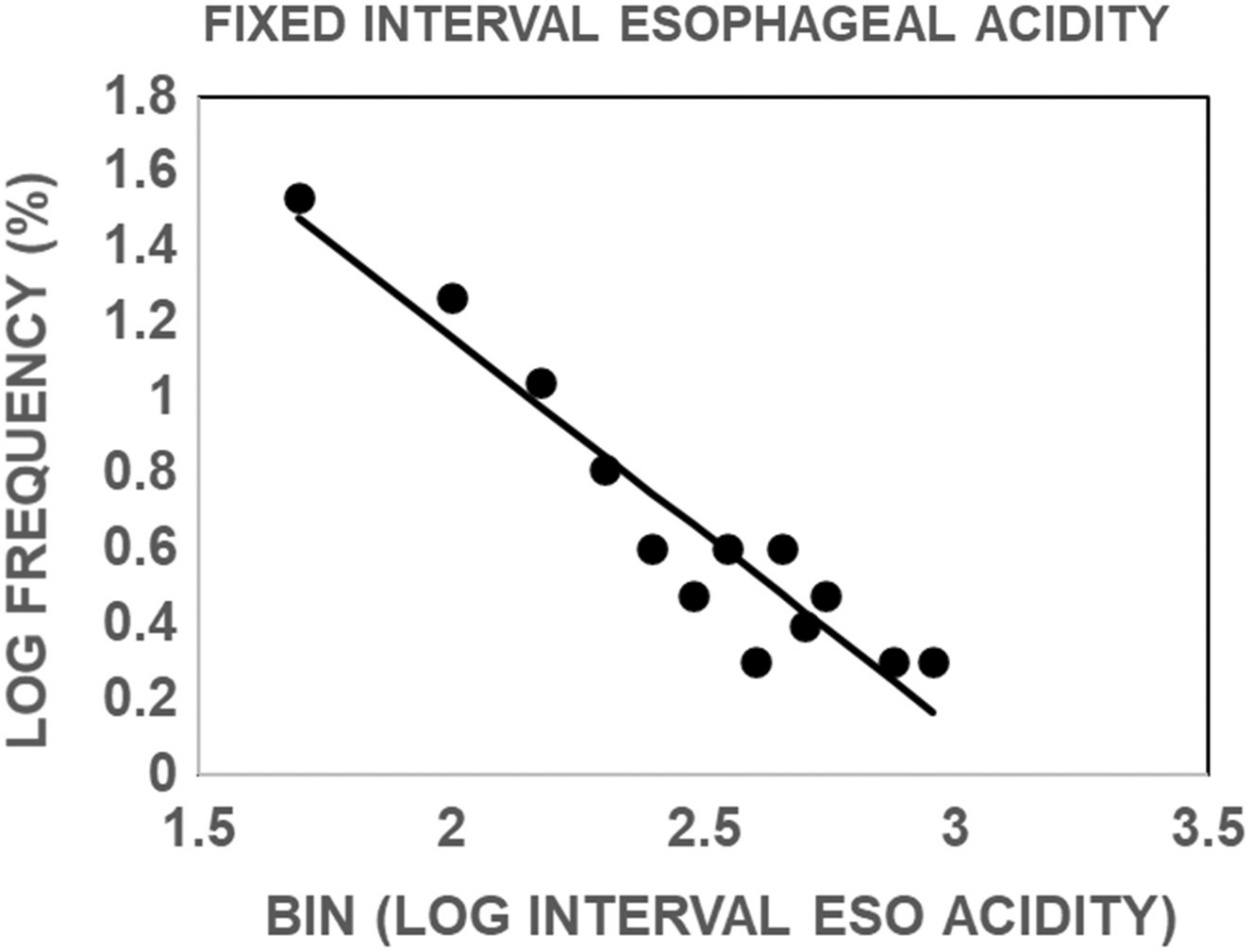
Distribution of values for interval esophageal acidity during a 60‐s interval preceding each of 476 symptoms. The solid line is the linear, least‐squares fit of the data and was significantly different from zero *p* < 0.0001 by an *F* test.

Figure 6 illustrates that the distribution of values of fixed interval esophageal acidity is similar to that for interval esophageal acidity shown in Figure 3 in that the lower the value of fixed interval esophageal acidity, the higher the probability of a symptom. These results also indicate that interval time is not an important determinant of the frequency distribution, and that when time is held constant the lower the value of fixed interval esophageal acidity, the higher the probability of a symptom.

**FIGURE 7:**
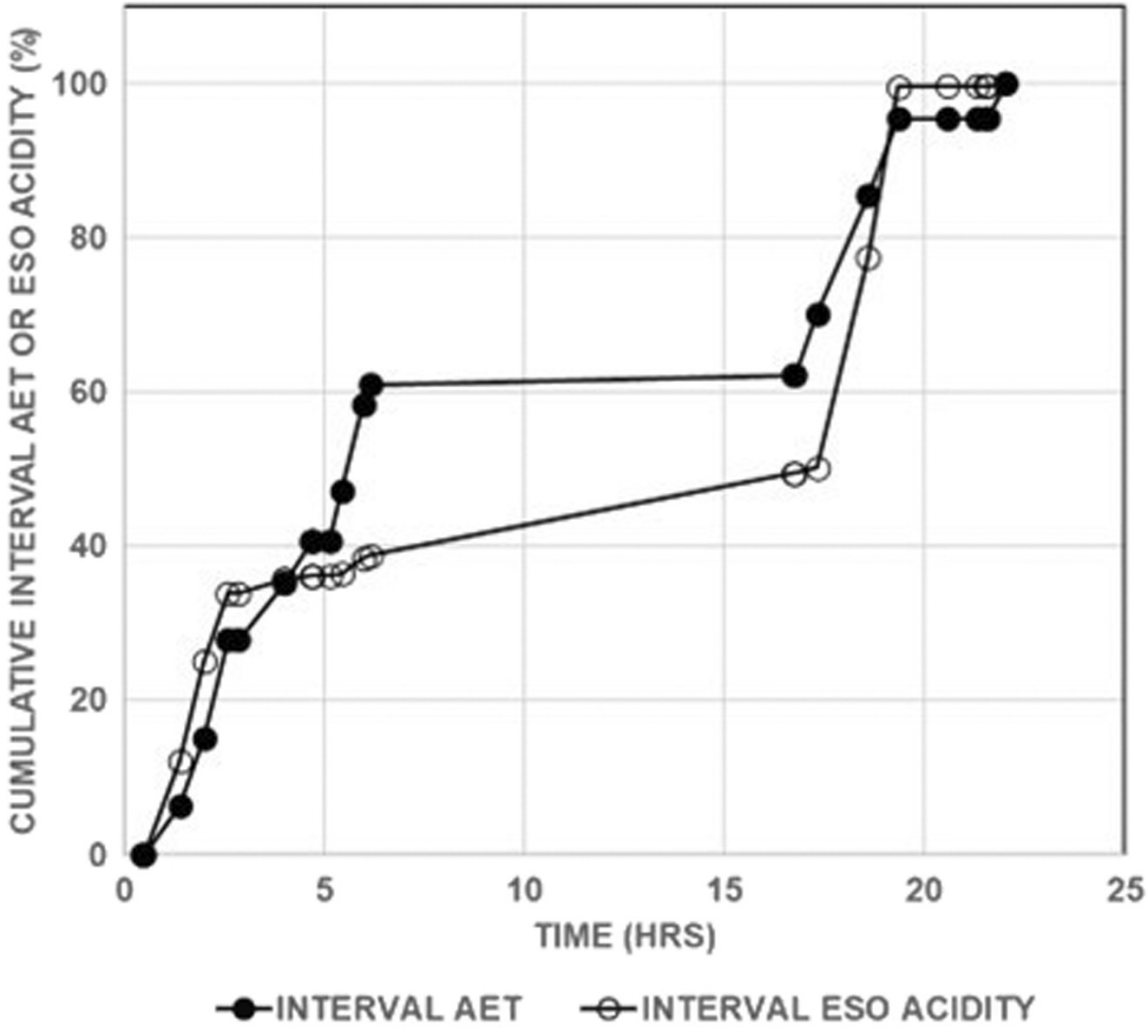
Cumulative esophageal acid exposure calculated for the nonerosive gastroesophageal reflux disease subject whose data are given in Figure 1 and Table 1. AET, esophageal acid exposure time.

Plotting cumulative esophageal acidity over time makes it possible to illustrate the increase in esophageal acid exposure with each successive symptom. The slope of the line that characterizes a series of symptoms (referred to as a segment) is a measure of esophageal acid exposure for the symptoms in that series, in that the steeper the slope, the higher the esophageal acid exposure. Figure 7 shows that for the NERD subject's pH recording illustrated in Figure 1, with both measures of esophageal acid exposure, there is an initial segment with a steep slope, followed by a segment with a shallow slope, followed by a segment with a steep slope, and finally followed by a segment with a shallow slope. Thus, this subject's symptoms occur in segments that are associated with alternating periods of high esophageal acid exposure and low esophageal acid exposure.

**FIGURE 8:**
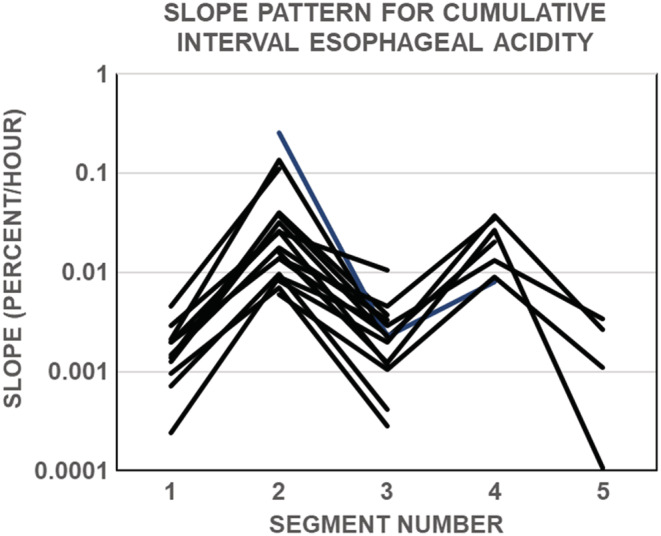
Slope patterns for cumulative interval esophageal acidity from 19 nonerosive gastroesophageal reflux disease subjects. Slopes were calculated using piecewise linear regression for each segment. Data from one subject are omitted because the subject had a single slope for the entire time course. If the pattern for a given subject began with a series of symptoms with a high slope, that subject's data were shifted to begin at the next segment.

Figure 8 illustrates that all NERD subjects except one showed a series of symptoms that occurred in periods of low esophageal acid exposure alternating with periods of high esophageal acid exposure. Similar results occurred with data for esophageal acid exposure time (not shown).

## 4 DISCUSSION

Nearly all previous analyses of relationships between GERD symptoms and esophageal acidity have used the SI or the SAP, which are limited to symptoms that are associated with esophageal reflux episodes (Gyawali et al., 2018; Kamal et al., 2020; Weiner et al., 1988; Weusten et al., 1994). Calculating esophageal acid exposure over the entire interval that precedes a symptom results in every symptom in every subject being associated with distinct values of interval esophageal acid exposure.

The present analyses using interval esophageal acidity and interval esophageal acid exposure time provide novel information regarding relationships between esophageal acidity and symptom frequency in symptomatic NERD subjects. Frequency distributions of values for interval esophageal acidity as well as interval esophageal exposure time showed a paradoxical relationship between the probability of a symptom and the value of interval esophageal acid exposure in that the lower the value of interval acidity the higher the probability of a symptom. This paradox of less esophageal acid and more symptoms was unanticipated, because nearly all published literature in the GERD field specifies or implies a direct relationship between esophageal acid and symptoms. For example, the occurrence of GERD symptoms has been reported to be associated with increased esophageal acid exposure time during the time before the symptom (Beedassy et al., 2000; Bredenoord et al., 2006) and reductions in esophageal acidity with gastric antisecretory agents such as PPIs reduce the occurrence of symptoms (Gyawali et al., 2018). The present findings indicate that it is unlikely that values for total esophageal acid exposure will provide meaningful information regarding symptom frequency in NERD subjects because some symptoms will be preceded by low values of esophageal acid and other symptoms will be preceded by high values of esophageal acid.

One possible explanation for the relationship between symptoms and low values of interval esophageal acid exposure is that “something else” besides esophageal acid, such as bile or pepsin, is responsible for the symptoms. Although perfusion of bile salts into the esophagus can induce heartburn in subjects with functional heartburn or normal subjects (Siddiqui et al., 2005), the presence of bile in the esophagus is associated with only a small percentage of symptoms in GERD subjects (Bredenoord, 2012). Pepsin can cause damage to esophageal mucosa in experimental settings (Bredenoord, 2012); however, there is no good evidence that pepsin is involved with symptoms in GERD subjects (Bredenoord, 2012). The presence of gas in reflux material (Bredenoord, 2012) as well as reflux material that reaches the upper esophagus can enhance the perception of GERD symptoms (Bredenoord, 2012). Neither of these phenomena, however, would be expected to occur with sufficient frequency to account for low values for interval esophageal acidity being associated with a high probability of GERD symptoms. It is also known that psychological factors can modify symptom perception in symptomatic GERD (Rubenstein et al., 2021), and possibly psychological factors amplify the esophageal sensitivity to low values of esophageal acid exposure.

Another possible explanation for the paradoxical finding is that the lower the value of esophageal acid exposure the higher the probability of symptoms comes from analyses of the time course of interval esophageal acidity in individual subjects. In individuals with symptomatic NERD, values for interval esophageal acidity tended to occur in clusters and in oscillating sequences of high and low interval acid exposure. Thus, a sequence of symptoms with low interval acid exposure could reflect high esophageal acid sensitivity, and the high acid exposure might reflect low esophageal acid sensitivity. The neurochemical mediators of symptoms produced by esophageal acid exposure have not been clearly established; however, the acid‐sensitive, transient receptor potential cation channel that is associated with the capsaicin or vanilloid receptor (TRPV1) is a possible candidate (Ang et al., 2008). Protein kinase C and nerve growth factor are both capable of sensitizing TRPV1 by lowering the activation threshold to hydrogen ions (Koivisto et al., 2022). Once activated, TRPV1 can undergo reversible desensitization (Koivisto et al., 2022). In addition, acid‐sensing ion channels are members of the voltage‐insensitive, amiloride‐sensitive degenerin family of cation channels that can be activated by protons (Waldmann & Lazdunski, 1998). These channels are upregulated in the esophageal mucosa from GERD patients and increased expression of ASCI correlates positively with symptom severity in GERD (Han et al., 2022). Studies in rats implicate ASCIs in visceral hypersensitivity mediated by vagal afferents in GERD (Han et al., 2022; Lennerz et al., 2007). Thus, it seems likely that neurons in esophageal mucosa play an important role in determining esophageal acid sensitivity. It is conceivable that with low esophageal acid exposure, nociceptive neurons in esophageal mucosa become sensitized to luminal acid. This sensitization is then followed by reversible desensitization of the neurons that, in turn, results in higher esophageal acid exposure being necessary to elicit symptoms. Since the desensitization is reversible, it eventually wanes or is overcome by renewed sensitization and the cycle repeats itself.

The association of symptoms with oscillating esophageal acid exposure might influence the response of symptoms in NERD subjects to gastric antisecretory agents such as PPIs. For example, if symptoms associated with low values of esophageal acid exposure are actually caused by “something else”, these symptoms might not be affected by a gastric antisecretory agent. Furthermore, if all symptoms are caused by esophageal acid exposure, symptoms associated with high values of esophageal acid might be less likely to be affected by a gastric antisecretory agent than symptoms associated with low values of esophageal acid.

NERD subjects have been found to have increased sensitivity to both chemical and mechanical stimuli (Bhalla et al., 2004; Howard et al., 1991; Rodriguez‐Stanley et al., 1999; Smith et al., 1989; Trimble et al., 1995). The present results indicate that NERD subjects may also have increased sensitivity to endogenous esophageal acid, and that this sensitivity oscillates.

There are some limitations to the present analyses. A major limitation is the lack of a causal explanation for the oscillating esophageal acid sensitivity in NERD subjects. I realize that no set of analyses can address all relevant questions, and others have pointed out that it may not be possible to correlate the behavior of each specific detail of a time series with a particular event (Fossion et al., 2020). It seems to me, however, that future attempts to identify the basis for the oscillating esophageal acid sensitivity in NERD subjects should have a high priority. There were also other limitations such as no standardized definition of a subject's unsatisfactory response to a gastric antisecretory agent. The impedance–pH recordings began at different times during the day and meals were not standardized with respect to time of the day or composition. The recumbent periods were not standardized. These features may have influenced important relationships among esophageal acidity and symptom frequency. Also, symptom severity was not assessed making it impossible to examine possible relationships between interval acidity or symptom frequency and symptom severity.

## CONFLICT OF INTEREST

Nothing to disclose.

## Appendix

**TABLE A1:** Standard values from impedance and pH monitoring for subjects in the present study

	S6	S7	S8	S12	S17	S19	S20	S29	S36	S39
Gender	F	M	F	M	F	M	F	M	M	M
Age (year)	74	23	56	52	53	22	73	33	30	32
Duration (h:min)										
Upright	12:42	8:54	16:57	10:26	10:22	11:36	10:50	15:50	13:32	10:34
Recumbent	6:48	10:22	8:38	9:49	9:45	7:59	10:17	9:01	9:53	12:33
Total	19:31	19:16	25:35	20:16	20.07	19:35	21:07	24:52	23:25	23:07
Acid exposure (%)										
Upright	14.6	9.5	11.0	12.7	11.3	8.6	2.1	10.1	19.9	24.6
Recumbent	1.4	4.5	30.8	0.0	3.2	8.8	22.8	0.2	19.5	29.5
Total	10.0	6.8	17.7	6.6	7.4	8.7	12.1	6.5	19.7	27.3
Reflux episodes (#)										
Acid	41	103	70	25	36	58	18	65	101	145
Non‐acid	9	29	18	6	15	10	7	24	4	38
Total	50	132	88	31	51	68	25	89	105	183
Symptom index (%)										
Hrtburn	80	65	76	80	83	76	40	78	80	72
Regurg	100	78	100	83	100	100			0	100
Chest pain	100	100		100			0	0		
Symptom association probability (%)										
Hrtburn	100	100	100	100	100	100	83	100	100	100
Regurg	100	100	99	100	0	0			0	78
Chest pain	92	83		100			0	0		

	S44	S46	S50	S53	S54	S55	S57	S58	S59	S60
Gender	F	M	F	M	F	F	M	F	F	F
Age (year)	63	60	53	39	32	44	54	31	68	45
Duration (h:min)										
Upright	10:50	9:59	7:13	9:26	6:39	16:41	11:48	11:40	12:29	14:52
Recumbent	10:12	8:25	11:41	10:44	11:27	4:53	8:34	9:14	9:01	6:08
Total	21:02	18:24	18:54	20:10	18:06	21.34	20:22	20:54	21:31	21:00
Acid exposure (%)										
Upright	37.2	3.1	28.2	38.7	1.4	13.8	11.5	4.2	2.8	13.2
Recumbent	47.9	46.1	40.8	17.0	12.2	0.0	0.5	10.8	12.3	15.6
Total	42.4	22.8	36.0	27.2	8.2	10.7	6.9	7.2	6.8	13.9
Reflux episodes (#)										
Acid	122	34	77	117	9	86	27	58	38	57
Non‐acid	32	4	8	12	12	9	27	2	35	60
Total	154	38	85	129	21	95	54	60	73	117
Symptom index (%)										
Hrtburn	89	93	100	89	58	97	38	0	50	55
Regurg	90	100	100		0	50	50		0	100
Chest pain	0			100			29			
Symptom association probability (%)										
Hrtburn	100	100	100	100	100	100	87	0	88	100
Regurg	99	93	98		0	0	0		0	97
Chest pain	0			97			55			

*Note*: Numbers at top of each column are subject numbers.

Abbreviations: Hrtburn, heartburn; Regurg, regurgitation.

## References

[phy215442-bib-0001] Ang, D. , Sifrim, D. , & Tack, J. (2008). Mechanisms of heartburn. Nature Clinical Practice Gastroenterology and Hepatology, 5, 383–391. 10.1038/ncpgasthep1160 18542113

[phy215442-bib-0002] Beedassy, A. , Katz, P. O. , Gruber, A. , Peghini, P. L. , & Castell, D. O. (2000). Prior sensitization of esophageal mucosa by acid reflux predisposes to reflux‐induced chest pain. Journal of Clinical Gastroenterology, 31, 121–124. 10993426 10.1097/00004836-200009000-00006

[phy215442-bib-0003] Bhalla, V. , Liu, J. , Puckett, J. L. , & Mittal, R. K. (2004). Symptom hypersensitivity to acid infusion is associated with hypersensitivity of esophageal contractility. American Journal of Physiology. Gastrointestinal and Liver Physiology, 287, G65–G71. 14977636 10.1152/ajpgi.00420.2003

[phy215442-bib-0004] Bredenoord, A. J. (2012). Mechanisms of reflux perception in gastroesophageal reflux disease: A review. The American Journal of Gastroenterology, 107, 8–15. 22218024 10.1038/ajg.2011.286

[phy215442-bib-0005] Bredenoord, A. J. , Weusten, B. L. A. M. , Curvers, W. L. , Timmer, R. , & Smout, A. J. P. M. (2006). Determinants of perception of heartburn and regurgitation. Gut, 55, 313–318. 16120760 10.1136/gut.2005.074690 PMC1856084

[phy215442-bib-0006] Fossion, R. , Saenz‐Burrola, A. , & Zapata‐Fonseca, L. (2020). On the stability and adaptability of human physiology: Gaussians meet heavy‐tailed distributions. Inter Disciplina, 8, 55–81.

[phy215442-bib-0007] Gardner, J. D. , Rodriguez‐Stanley, S. , & Robinson, M. (2001). Integrated acidity and the pathophysiology of GERD. The American Journal of Gastroenterology, 96, 1363–1370. 11374669 10.1111/j.1572-0241.2001.03790.x

[phy215442-bib-0008] Gardner, J. D. , Young, W. , Sloan, S. , Robinson, M. , & Miner, P. B., Jr. (2005). The fractal nature of human gastro‐oesophageal disease. Alimentary Pharmacology & Therapeutics, 22, 823–830. 16225491 10.1111/j.1365-2036.2005.02665.x

[phy215442-bib-0009] Gyawali, C. P. , Kahrilas, P. J. , Savarino, E. , Zerbib, F. , Mion, F. , Smout, A. J. P. M. , Vaezi, M. , Sifrim, D. , Fox, M. R. , Vela, M. F. , Tutuian, R. , Tack, J. , Bredenoord, A. J. , Pandofilno, J. , & Roman, S. (2018). Modern diagnosis of GERD: The Lyon consensus. Gut, 67, 1351–1362. 29437910 10.1136/gutjnl-2017-314722 PMC6031267

[phy215442-bib-0010] Han, X. , Zhang, Y. , Lee, A. , Li, Z. , Gao, J. , Wu, X. , Zhao, J. , Wang, H. , Chen, D. , Zou, D. , & Owyang, C. (2022). Upregulation of acid sensing ion channels is associated with esophageal hypersensitivity in GERD. The FASEB Journal, 36, e22083. 34918385 10.1096/fj.202100606R PMC8715981

[phy215442-bib-0011] Howard, P. J. , Maher, L. , Pryde, A. , & Heading, R. C. (1991). Symptomatic gastroesophageal reflux, abnormal oesophageal acid exposure, and mucosal acid sensitivity are three separate, though related aspects of gastro‐oesophageal reflux disease. Gut, 32, 128–132. 1864528 10.1136/gut.32.2.128 PMC1378792

[phy215442-bib-0012] Kamal, A. N. , Clarke, J. O. , Oors, J. M. , Smout, A. J. , & Bredenoord, A. J. (2020). The role of symptom association analysis in gastroesophageal reflux testing. The American Journal of Gastroenterology, 115, 1950–1959. 32740077 10.14309/ajg.0000000000000754

[phy215442-bib-0013] Koivisto, A.‐P. , Belvisi, M. C. , Gaudet, R. , & Szallasi, A. (2022). Advances in TRP channel drug discovery: From target validation to clinical studies. Nature Reviews Drug Discovery, 21, 41–59. 34526696 10.1038/s41573-021-00268-4 PMC8442523

[phy215442-bib-0014] Lennerz, J. K. M. , Dentsch, C. , Bernardini, N. , Hummel, T. , Neuhuber, W. L. , & Reeh, P. W. (2007). Electrophysiological characterization of vagal afferents relevant to mucosal nociception in the rat upper oesophagus. The Journal of Physiology, 582, 229–242. 17478536 10.1113/jphysiol.2007.130823 PMC2075303

[phy215442-bib-0015] Rodriguez‐Stanley, S. , Robinson, M. , Earnest, D. L. , Greenwood‐Van Meerveld, B. , & Miner, P. B., Jr. (1999). Esophageal hypersensitivity may be a major cause of heartburn. The American Journal of Gastroenterology, 94, 628–631. 10086642 10.1111/j.1572-0241.1999.00925.x

[phy215442-bib-0016] Rubenstein, J. H. , Jianh, L. , Kurlander, J. E. , Chen, J. , Metko, V. , Khodadost, M. , Nofz, K. , & Raghunathan, T. (2021). Incomplete response of gastroesophageal reflux symptoms poorly predicts erosive esophagitis or Barrett's esophagus. Gastroenterology, 19, 2284–2292. 10.1016/j.cgh.2020.08.044 32835843

[phy215442-bib-0017] Siddiqui, A. , Rodriguez‐Stanley, S. , Zubaidi, S. , & Miner, P. B., Jr. (2005). Esophageal visceral sensitivity to bile salts inpatients with functional heartburn and in healthy control subjects. Digestive Diseases and Sciences, 50, 81–85. 15712642 10.1007/s10620-005-1282-0

[phy215442-bib-0018] Smith, J. L. , Operkun, A. R. , Larkai, E. , & Graham, D. Y. (1989). Sensitivity of the esophageal mucosa to pH in gastroesophageal reflux disease. Gastroenterology, 96, 683–689. 2914634

[phy215442-bib-0019] Trimble, K. C. , Pryde, A. , & Heading, R. C. (1995). Lowered oesophageal sensory thresholds in patients with symptomatic but not excess gastro‐oesophageal reflux. Evidence for a spectrum of visceral sensitivity in GORD. Gut, 37, 7–12. 7672684 10.1136/gut.37.1.7 PMC1382759

[phy215442-bib-0020] Waldmann, R. , & Lazdunski, M. (1998). H(+)‐gated cation channels: Neuronal acid sensors in the NaC/DEG family of ion channels. Current Opinion in Neurobiology, 8, 418–424. 9687356 10.1016/s0959-4388(98)80070-6

[phy215442-bib-0021] Weiner, G. J. , Richter, J. E. , Copper, J. B. , Wu, W. C. , & Castell, D. O. (1988). The symptom index: A clinically important parameter of 24‐hour esophageal pH monitoring. The American Journal of Gastroenterology, 83, 358–361. 3348191

[phy215442-bib-0022] Weusten, B. L. , Roelofs, J. M. , Akkermans, L. M. , Van Berge‐Henegouwen, G. P. , & Smout, A. J. (1994). The symptom‐association probability: An improved method for symptom analysis of 24‐hour esophageal pH data. Gastroenterology, 107, 1741–1745. 7958686 10.1016/0016-5085(94)90815-x

